# Supplementary material for: Heparin Blocks the Inhibition of Tissue Kallikrein 1 by Kallistatin through Electrostatic Repulsion
Source: Biomolecules. 2020 May 28;10(6):828. doi: 10.3390/biom10060828 (PMC7356578; doi:10.3390/biom10060828)
Supplement: Supplementary file 1 [file biomolecules-10-00828-s001.pdf]

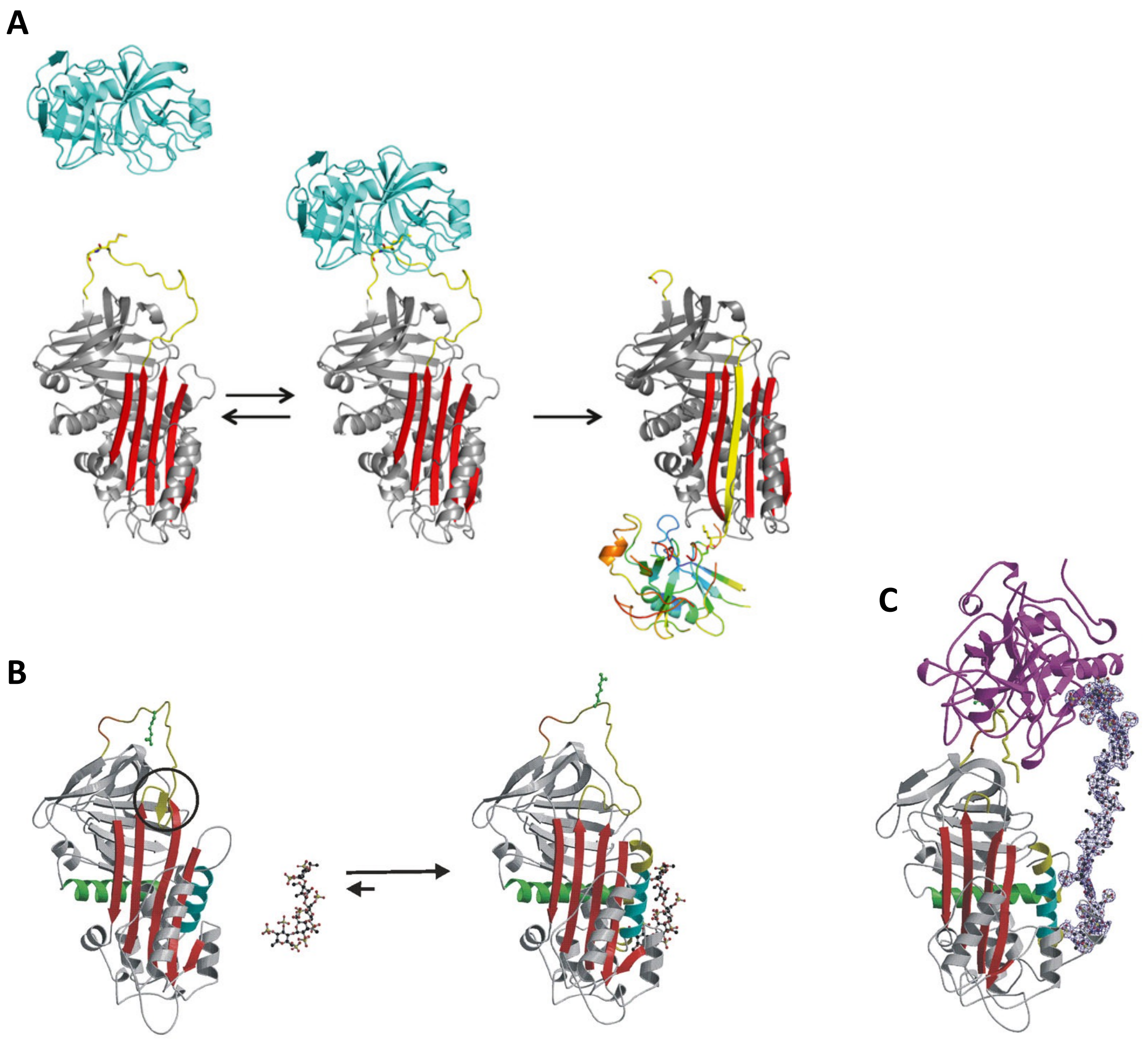

Figure S1. (A) Serpin structure and mechanism of protease inhibition. The common serpin mechanism can be described minimally by three panels. Left shows the protease (top, cyan) and the serpin (bottom, grey with RCL in yellow and P1–P1′ as sticks, and β-sheet A in red) before interacting with one another. The central panel depicts a typical Michaelis complex. The right panel shows the final serpin-protease complex, with the protease translocated to the opposite pole of the serpin and covalently linked by an ester bond (acylenzyme intermediate). The protease is coloured according to B-factor, with orange and red indicating disorder. About 40% of the protease is missing in the structure due to the reversal of the zymogen activation mechanism. **(B)** The binding of the specific heparin pentasaccharide to antithrombin induces a global conformational change involving the expulsion of the hinge region (circled) of the reactive center loop (RCL, yellow) from the central β-sheet A (red), and extension (yellow) of the A and D helices (green and cyan, respectively). The expulsion of the hinge region increases the flexibility of the RCL and liberates the P1 Arg (green ball-and-stick). The flexibility of the C-terminal portion of the RCL (P′ side) is limited, despite a three-residue insertion (orange), owing to a tight hydrogen-bonded turn. **(C)** Of the hinge region of antithrombin in its complex with thrombin and heparin (yellow) reveals the insertion of P15 Gly into β-sheet A, and a larger opening between strands 3 and 5A than seen for pentasaccharide- bound antithrombin alone (gray). It has been shown that high-affinity binding is not inconsistent with a native-like hinge conformation, as demonstrated by the structure in PDB entry 1NQ9 (brown).

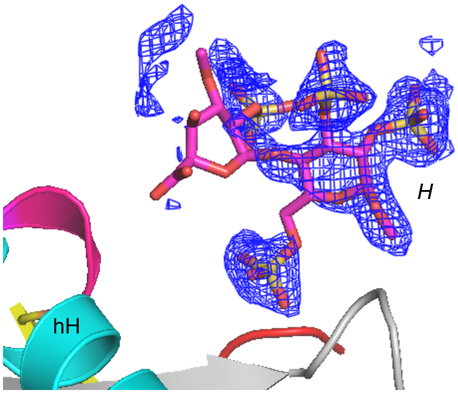

Figure S2. A polder map showing the electron density of heparin saccharides in the structure. The polder map covering the two heparin saccharides in the crystal structure was calculated in Phenix and the density contoured at 3-sigma is shown here. There are clear electron density showing four sulphonate groups The map coefficient (CC) calculated with the ligand is 0.825, which is significantly larger than the map coefficient calculated without the ligand (CC=0.4727). This indicates that heparin is present in the structure.

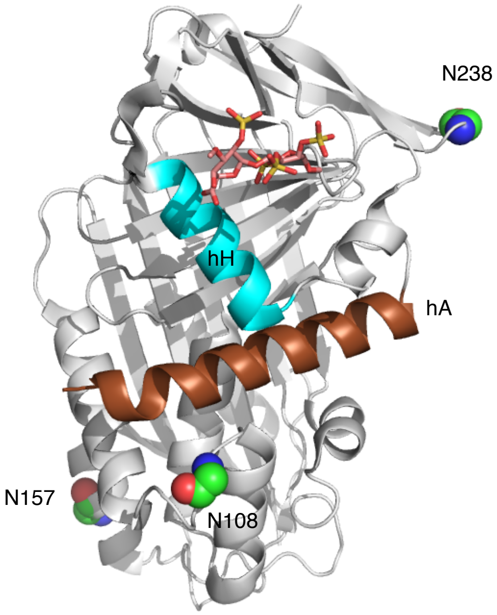

Figure S3. Potential glycosylation sites of human kallistatin. Three potential glycosylation sites were shown in spheres. N108 is located between helix C and D, N157 is located on hE and N238 is located in the gate region. The heparin saccharides (pink) are shown in sticks with Helix A coloured in brown and helix H in cyan.

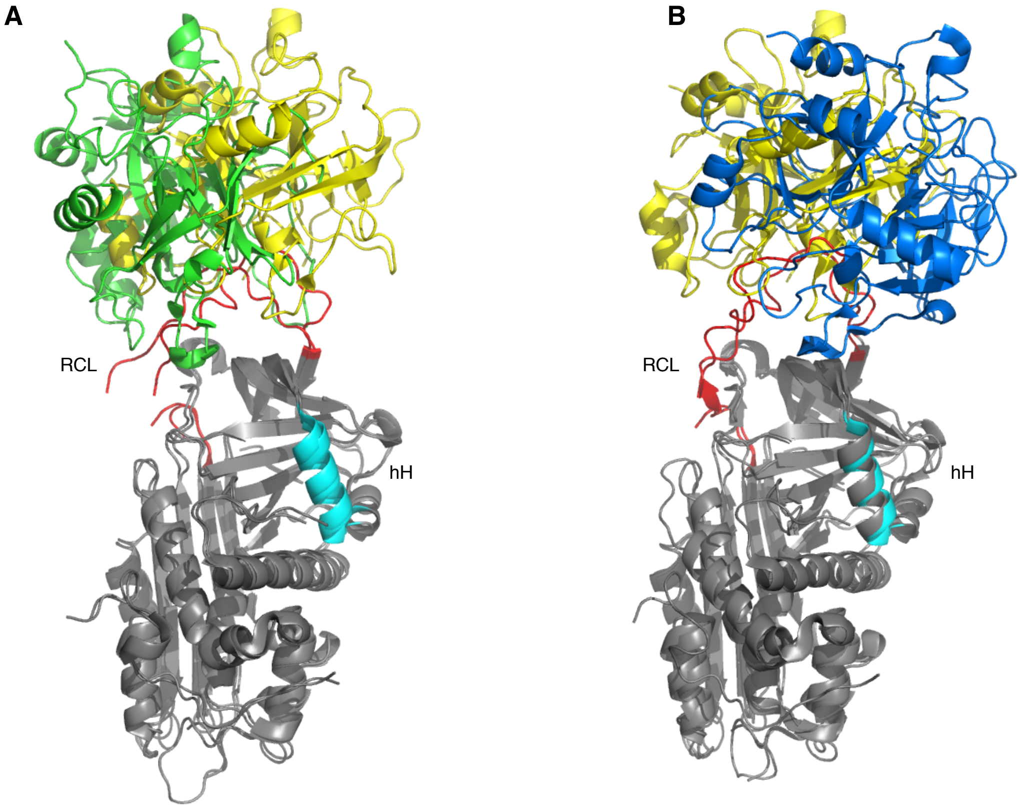

Two antithrombin-thrombin Michaelis complexes

Antithrombin-thrombin and PCI-thrombin Michaelis complexes

Figure S4. Overlaid structures of the Michaelis complex of thrombin with antithrombin or PCI. **(A)** Two antithrombin-thrombin Michaelis complex structures were superposed by antithrombin positions showing different orientations of thrombin. Thrombin in PDB 1S5R is in yellow and thrombin in PDB 1TB6 is in green. The helix H is colored in cyan and the reactive center loop of antithrombin is colored in red. **(B)** The antithrombin-thrombin Michaelis complex structure (PDB 1S5R) was superposed with that of the PCI-thrombin complex (PDB 3B9F) by the serpin positions. Thrombin of PCI-thrombin complex is colored marine. This shows that the helix H of serpin is close to the body of the protease, especially in the PCI-thrombin complex structure. It is expected that KLK1 would form similar Michaelis complex with kallistatin where hH of kallistatin is close proximity of KLK1 surface.

Table S1. Second order association rate constants for the interaction between kallistatin and KLK1/KLK1-70Lm/KLK1-90Lm. The apparent second order rate constant (k_{app}) was determined from the slope of the linear plot of k_{obs} versus the inhibitor concentration. KLK1, KLK1-70Lm and KLK1-90Lm was mixed with different amount of kallistatin and linear regression analysis of the decrease in protease activity with the concentration of kallistatin yielded the stoichiometry of inhibition (SI) as the intercept on the abscissa. The product of k_{app} × SI represents the second order rate constant (k_2). Each experiment was repeated three times with mean ± SD calculated.

**KLK1**	**rKAL**		
	**k_{app} ($M^{-1}s^{-1}$)**	**SI**	**k_{app} × SI ($M^{-1}s^{-1}$)**
KLK1	2.4 ± 0.1 × 10^3	1.4 ± 0.1	3.4 × 10^3
KLK1-70Lm	1.2 ± 0.1 × 10^3	1.5 ± 0.1	1.8 × 10^3
KLK1-90Lm	1.3 ± 0.1 × 10^2	1.6 ± 0.1	2.0 × 10^2
